# Recognition of V^3+^/V^4+^/V^5+^ Multielectron Reactions in Na_3_V(PO_4_)_2_: A Potential High Energy Density Cathode for Sodium-Ion Batteries

**DOI:** 10.3390/molecules25041000

**Published:** 2020-02-24

**Authors:** Rui Liu, Ziteng Liang, Yuxuan Xiang, Weimin Zhao, Haodong Liu, Yan Chen, Ke An, Yong Yang

**Affiliations:** 1 School of Materials Science and Engineering, Shandong University of Science and Technology, Qingdao 266590, China; liurui@sdust.edu.cn; 2 Collaborative Innovation Center of Chemistry for Energy Materials, State Key Laboratory for Physical Chemistry of Solid Surface, Department of Chemistry, College of Chemistry and Chemical Engineering, Xiamen University, Xiamen 361005, Chinanuaaxyx@163.com (Y.X.); 3 College of Chemical Engineering and Safety, Binzhou University, Binzhou 256603, China; zhaoweimin137@sina.com; 4 Department of NanoEngineering, University of California San Diego, 9500 Gilman Drive, La Jolla, CA 92093, USA; 5 Neutron Scattering Division, Oak Ridge National Laboratory, Oak Ridge, TN 37830, USA; cheny1@ornl.gov (Y.C.); kean@ornl.gov (K.A.); 6 School of Energy Research, Xiamen University, Xiamen 361005, China

**Keywords:** polyanion, energy density, multielectron reaction, solid-state NMR

## Abstract

Na_3_V(PO_4_)_2_ was reported recently as a novel cathode material with high theoretical energy density for Sodium-ion batteries (SIBs). However, whether V^3+^/V^4+^/V^5+^ multielectron reactions can be realized during the charging process is still an open question. In this work, Na_3_V(PO_4_)_2_ is synthesized by using a solid-state method. Its atomic composition and crystal structure are verified by X-ray diffraction (XRD) and neutron diffraction (ND) joint refinement. The electrochemical performance of Na_3_V(PO_4_)_2_ is evaluated in two different voltage windows, namely 2.5–3.8 and 2.5–4.3 V. ^51^V solid-state NMR (ssNMR) results disclose the presence of V^5+^ in Na_2−*x*_V(PO_4_)_2_ when charging Na_3_V(PO_4_)_2_ to 4.3 V, confirming Na_3_V(PO_4_)_2_ is a potential high energy density cathode through realization of V^3+^/V^4+^/V^5+^ multielectron reactions.

## 1. Introduction

Large-scale energy storage systems (ESSs) that are used in renewable solar and wind energy systems and smart grids have received great attention due to increasing energy demands [1,2,3]. The low cost and inexhaustible and ubiquitous sodium resources make Sodium-ion batteries (SIBs) an attractive and promising candidate for ESSs [3,4]. In this case, many types of compounds including layered oxides [5,6,7], polyanionic frameworks [8,9,10,11] and hexacyanoferrates [12,13,14] have been explored as cathode materials for SIBs. Among them, polyanion-based compounds have attracted extensive interest due to their excellent cycling stability, high safety, and adjustable voltage [8,9,10]. However, the specific capacity and energy density of polyanion-based compounds are generally lower than the layered transition metal oxides [15,16]. More specifically, the energy density of polyanion-type materials is usually lower than 500 Wh/kg [15].

Recently we have reviewed the progress of multielectron reactions in polyanionic materials and concluded that exploring multielectron reactions in polyanionic cathodes could substantially improve the energy density by increasing both the reacting electron number and the voltage of cathodes according to Equation (1) [17]:

(1) $$\begin{array}{c} E=QV\\ =26800\frac{nV}{M}(\mathrm{Wh}/\mathrm{kg}) \end{array}$$

where *Q* is the specific capacity, *V* is the voltage vs. Na^+^/Na in this work, *n* is the number of electrons involved in the reaction, and *M* is the molecular weight of the material. We have further proposed that V^3+^/V^4+^/V^5+^ and Mn^2+^/Mn^3+^/Mn^4+^ redox couples are readily accessible in many polyanionic cathodes.

**Figure 1:**
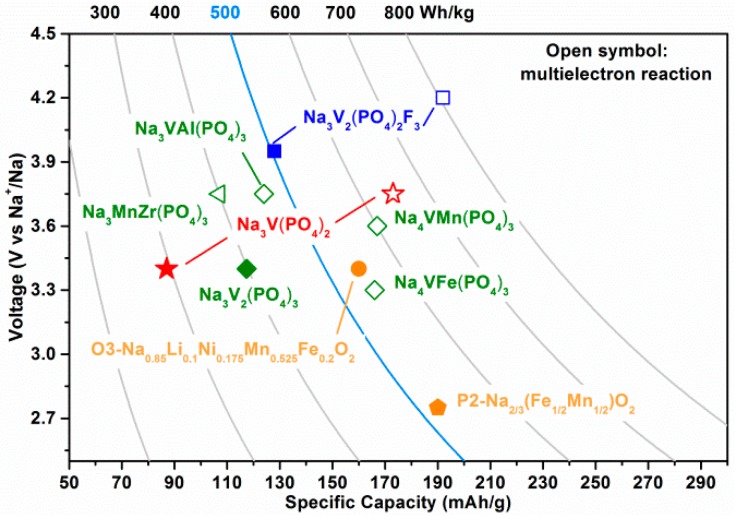
Operation voltages versus specific capacities of cathode materials for sodium-ion batteries.

Figure 1 shows polyanion-type cathodes with multielectron reactions which are plotted on the basis of Table S1 [18,19,20,21,22,23,24,25,26,27]. Among them, the well-known NASICON (Na superionic conductors)-type Na_3_V_2_(PO_4_)_3_ exhibits one V^3+^/V^4+^ redox couple at 3.4 V due to two Na (in the Na(2) site) extraction while the third Na in the Na(1) site could not be extracted in a common voltage range [28,29,30]. Consequently, a V/*M*^3+^ (*M*^3+^ = Fe^3+^ [31], Al^3+^ [26,32], Cr^3+^ [25,33], etc.) replacement could improve the energy density of Na_3_V_2_(PO_4_)_3_-based cathodes by introducing a high voltage plateau (~4.1 V) through the activation of a V^4+^/V^5+^ reaction. Our recent work has testified to the reversible V^3+^/V^4+^/V^5+^ reactions through ex situ X-ray absorption near edge structure (XANES) and ^51^V solid-state NMR (ssNMR) [25]. In addition, Goodenough et al. reported that reversible Mn^2+^/Mn^3+^/Mn^4+^ reactions could also be accessed in Na_3_Mn*M*(PO_4_)_3_ (*M*^4+^ = Ti^4+^ [23], Zr^4+^ [24], etc.) and thus show slightly enhanced energy density compared to the Na_3_V_2_(PO_4_)_3_. The multielectron reactions in Na_3_Mn*M*(PO_4_)_3_ are proved by X-ray photoelectron spectroscopy (XPS) at different states of charge [24]. However, the energy density of the above-mentioned cathodes is not improved much. Recently, V/*M*^2+^ (Fe^2+^ [18], Mn^2+^ [19,20], etc.) substitution was proven to improve the energy density of Na_4_V*M*(PO_4_)_3_ up to higher than 500 Wh/kg by introducing excess Na and new redox couples (Fe^2+^Fe/^3+^ or Mn^2+^/Mn^3+^/Mn^4+^) in addition to V^3+^/V^4+^/V^5+^ reactions. Unfortunately, they exhibit quick capacity loss and high structural irreversibility. Other than NASICON-type cathodes, Na_3_V_2_(PO_4_)_2_F_3_ shows an energy density of ~506 Wh/kg with two Na extraction and the oxidation of V^3+^ to V^4+^ below 4.3 V [34,35]. Very recently, Tarascon et al. reported that the third Na in Na_3_V_2_(PO_4_)_2_F_3_ can be extracted at ~4.7 V, thus resulting in a high theoretical energy density of 810 Wh/kg [21,22].

Na_3_V(PO_4_)_2_ was recently reported as a novel cathode material with a theoretical capacity of 173 mAh/g and two voltage plateaus at ca. 3.6 and 4.0 V, leading to a theoretical energy density of 657 Wh/kg [36,37]. The low-voltage plateau can be ascribed to the oxidation of V^3+^ to V^4+^ on the basis of V K-edge XANES spectra, which corresponds to one Na extraction [37]. However, the high-voltage plateau only shows in the first charge while disappeared in the following charge/discharge process. Moreover, only small changes are observed in this region from in situ XRD patterns, thus possible electrolyte decomposition cannot be ruled out [36]. Consequently, whether this 4.0 V plateau is ascribed to a V^4+^/V^5+^ reaction remains an open question.

^51^V ssNMR is a reliable method to ensure the presence of V^5+^, because only signals of V^5+^ compounds without localized *d* electron are visible when using standard NMR methods [25,35,38]. For instance, Croguennec et al. revealed a charge disproportionation of two V^4+^ ions into V^3+^ and V^5+^ occurs in NaV_2_(PO_4_)_2_F_3_ by using ^51^V ssNMR, which confirmed the presence of V^5+^ in NaV_2_(PO_4_)_2_F_3_ [35]. As mentioned above, we have also recognized V^5+^ in Na_2−*x*_VCr(PO_4_)_3_ through ^51^V ssNMR and further disclosed the V^3+^/V^4+^/V^5+^ multielectron reactions [25]. In this work, we revealed the presence of V^5+^ in Na_2−*x*_V(PO_4_)_2_ through ^51^V ssNMR. This is conclusive evidence that Na_3_V(PO_4_)_2_ is a potential high energy cathode with a high theoretical energy density of 657 Wh/kg.

## 2. Results

**Figure 2:**
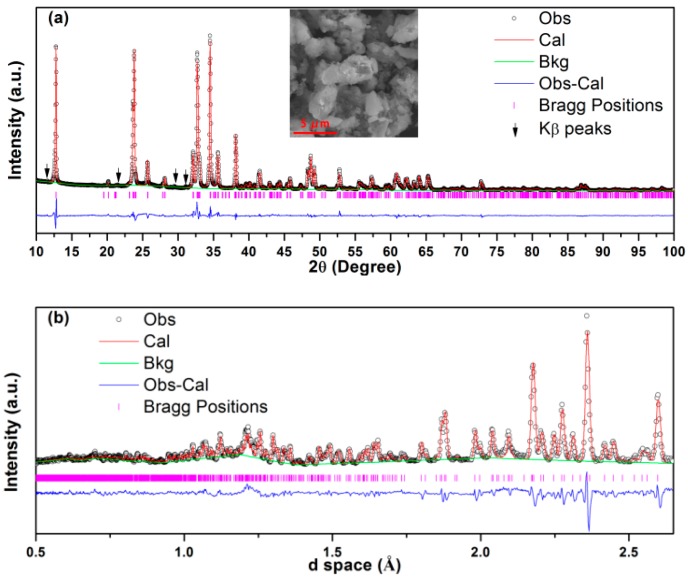
Combined Rietveld refinement of the (**a**) XRD (R_wp_ = 8.04%, R_p_ = 6.20%) and (**b**) neutron diffraction (ND) (R_wp_ = 6.82%,R_p_ = 5.39%) patterns of Na_3_V(PO_4_)_2_. Arrows in the XRD pattern indicate residual Cu K_β_ peaks caused by the diffractometer. The overall R_wp_ and R_p_ are 7.45% and 5.73%, respectively.

Recently, Kang and Masquelier et al. have obtained Na_3_V(PO_4_)_2_ almost at the same time. The crystal structure consists of a C2/c symmetry with a monoclinic system according to single crystal XRD [36,37]. Here we synthesized Na_3_V(PO_4_)_2_/C using a solid-state method and the atomic ratio of Na:P and V:P is determined to be 1.50 and 0.53 using inductively coupled plasma (ICP), respectively, which fits well with their theoretical values. Moreover, the structure of the product was further analyzed by XRD and neutron diffraction (ND). The combined Rietveld XRD and ND were carried out using the monoclinic structural model reported in [36,37], as shown in Figure 2. The structure of Na_3_V(PO_4_)_2_ is a C2/c symmetry with cell parameters of *a* = 9.09149(16) Å, *b* = 5.03480(10) Å, *c* = 13.86207(20) Å, *β* = 91.2456(16), and *V* = 634.37 Å^3^, which is in good agreement with the literature [36,37]. The detailed structure information is summarized in Table S2. Part of the bond length and angle were calculated based the obtained structure and listed in Table S3. It is worth to note, that the V–O bond length and P–O bond length fit well with the reported VO_6_ and PO_4_ results, respectively [11].

**Figure 3:**
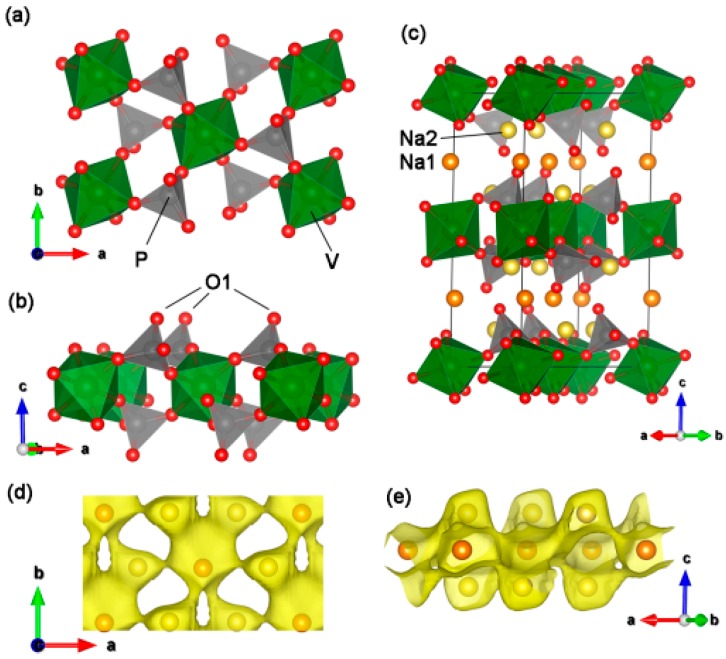
(**a**–**c**) Structural illustration and (**d**,**e**) BVS map of layered Na_3_V(PO_4_)_2_.

Figure 3 shows the schematic crystal structure of Na_3_V(PO_4_)_2_ based on the obtained structural information. The framework of Na_3_V(PO_4_)_2_ is built from VO_6_ octahedra and PO_4_ tetrahedra units, as shown in Figure 3a. Each VO_6_ octahedra connects with six PO_4_ tetrahedra and each PO_4_ tetrahedra connects to three VO_6_ octahedra, all in a corner-sharing mode, to form [V_5_(PO_4_)_6_] units. It is worth noting that one oxygen atom (O1) in the PO_4_ tetrahedra does not attach to the VO_6_ octahedra, as shown Figure 3b. Overall, the [V_5_(PO_4_)_6_] units are interconnected to form infinite slabs of [V_2_(PO_4_)_4_]_∞_ in the *ab* plane and further stack along the *c* direction to form a layered V(PO_4_)_2_ framework. There are two different oxygen environment interstitial sites in the layered V(PO_4_)_2_ framework: Na1 with six fold coordination and Na2 with eight fold coordination. Na1 and Na2 construct Na layers, which stack with V(PO_4_)_2_ layers alternatively along the *c* direction to form the crystal, as can be seen in Figure 3c. Figure S1 (Supplementary Materials) further demonstrates that Na1 and Na2 locate in line with V and P atoms along the *c* axis, respectively. Moreover, the bond valence sum (BVS) map shown in Figure 3d,e implies an evident 2D diffusion pathway of Na^+^ in the structure [39].

The inserted SEM image in Figure 2a and Figure S2 shows that the shape of the product is irregular with the particle size from several to tens of micrometers. Figure S3 shows nearly the same XANES edge position and pre-edge features of Na_3_V(PO_4_)_2_ and Na_3_VCr(PO_4_)_3_, in which the valance of the vanadium ion is +3, indicating that the oxidation state of the vanadium ion in Na_3_V(PO_4_)_2_ is +3 [25].

**Figure 4:**
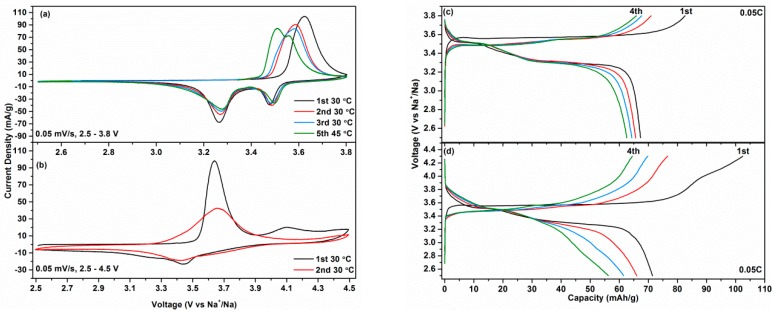
Cyclic voltammetry (CV) (**a**,**b**) and charge/discharge (**c**,**d**) curves of the Na_3_V(PO_4_)_2_ cathode.

The electrochemical performances of Na_3_V(PO_4_)_2_ as a Na insertion host compound were evaluated by cyclic voltammetry (CV) at a scan rate of 0.05 mV/s, as shown in Figure 4. The major CV features are the one anodic peak and two cathodic peaks during the first cycle in the voltage range of 2.5–3.8 V when tested at 30 °C. The anode peak shifts slightly toward a lower voltage in the second cycle and then keeps at the same position in the third cycle, meaning a good stability of the structure during Na (de)insertion. Besides, it is noted that the anodic peaks should be composited of two peaks according to the asymmetric feature. Indeed, the anodic peak splits into two peaks at 45 °C, indicating the sluggish kinetics of the low-voltage reaction. The quasi-open circuit voltage (QOCV) curve in Figure S4 further shows the sluggish kinetics of the low-voltage reaction. Masquelier et al. have also observed this phenomenon [36]. An additional anodic peak at ~4.1 V is observed from the CV curve when extending the voltage from 3.8 to 4.5 V, as shown in Figure 4b, implying another redox couple is activated. However, the extended voltage range results in inferior reversibility, as indicated by the decreased intensities of the anodic and cathodic peaks.

Figure 4c displays galvanostatic charge/discharge profiles in the voltage range of 2.5–3.8 V at rates of C/20. Notably, a capacity of 82.6 mAh/g can be obtained in the first charge with a relatively flat voltage plateau at ca. 3.56 V vs. Na^+^/Na, which is almost equal to 1 Na deinsertion. The discharge curve shows two plateaus at 3.51 and 3.31 V, resulting in a capacity of 67.3 mAh/g, corresponding to an initial coulombic efficiency of 81.5%. Furthermore, two plateaus can be seen from the charge curve in the following cycles, which is consistent with the CV results. An additional voltage plateau at ~4.1 V can be seen once the cut-off voltage becomes 4.3 V, as shown in Figure 4d. The charge capacity is 102.4 mAh/g, corresponding to a 1.2 Na deinsertion. Unfortunately, the discharge capacity is 71.4 mAh/g, which is only slightly higher than the discharge capacity of Na_3_V(PO_4_)_2_ with a narrower voltage window. Moreover, from Figure S5 we conclude that the capacity of Na_3_V(PO_4_)_2_ drops faster in the voltage window of 2.5–4.3 V than 2.5–3.8 V.

**Figure 5:**
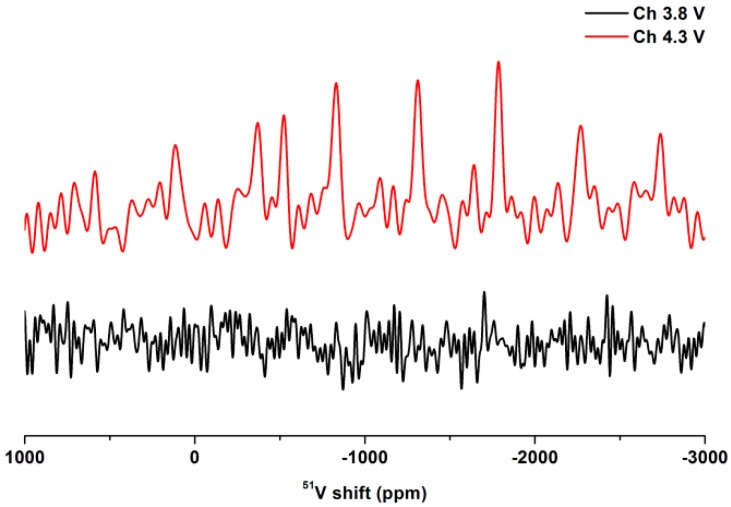
Ex situ ^51^V solid-state NMR (ssNMR) of Na_3_V(PO_4_)_2_ charged to different voltages in the first cycle.

Ex situ ^51^V ssNMR was carried out to recognize V^5+^ in Na_2−*x*_V(PO_4_)_2_ for testifying V^3+^/V^4+^/V^5+^ multielectron reactions of Na_3_V(PO_4_)_2_ when charging to 4.3 V. As shown in Figure 5, obvious peaks can be seen from the ^51^V NMR spectrum when charging Na_3_V(PO_4_)_2_ to 4.3 V. Because standard NMR methods only detect V^5+^, which possesses zero localized *d* electrons, the presence of V^5+^ in Na_2-*x*_V(PO_4_)_2_ is therefore proved. As a comparison, there is only noise signal in the ^51^V ssNMR spectrum by charging Na_3_V(PO_4_)_2_ to 3.8 V (i.e., Na_2_V(PO_4_)_2_), indicating that the V^5+^ ion is absent. In fact, Kang et al. have confirmed that the oxidation state is +4 in Na_2_V(PO_4_)_2_ through V K-edge XANES [37]. Consequently, Na_3_V(PO_4_)_2_ is demonstrated to be a potential high energy density cathode (657 Wh/kg) with V^3+^/V^4+^/V^5+^ multielectron reactions, albeit it displays inferior reversibility and cyclic stability. The capacity degradation of Na_3_V(PO_4_)_2_ is possibly caused by the gliding of V(PO_4_)_2_ slabs [6,7], large volume change [36,37], and collapsing of the framework [17] during Na deintercalation. These adverse effects commonly exist in layered transition metal oxide cathodes, which are mainly attributed to the local distortion caused by a drastic change of ion size and electrostatic repulsion between two slabs due to Na deintercalation [6,7]. It should be noted that although Na_3_V(PO_4_)_2_ shows unsatisfactory electrochemical performance at present, this work would attract lots of research interests to Na_3_V(PO_4_)_2_ due to its high theoretical energy density based on V^3+^/V^4+^/V^5+^ multielectron reactions. The theoretical energy density is expected to be realized by combining a better understanding of the working mechanism and further optimization of the material (e.g., a doping method which is frequently used for the layered transition metal cathodes [6,7]).

## 3. Discussion

In summary, as a cathode material for SIBs, Na_3_V(PO_4_)_2_ could reversibility uptake 1 Na at ~3.4 V with a V^3+^/V^4+^ reaction. Additional Na could be extracted at around 4.0 V when extending the upper cut-off voltage limit to 4.3 V. ^51^V ssNMR further revealed that the high voltage plateau could be ascribed to V^4+^/V^5+^ reactions. Consequently, Na_3_V(PO_4_)_2_ can potentially deliver two electrons through V^3+^/V^4+^/V^5+^ reactions, thus resulting in a high theoretical energy density of 657 Wh/kg, which outperforms most of the known polyanion and layered oxides. Albeit the reversibility and the observed energy density are still far from the theoretical value, we believe this material is worth further investigation due to its potential high energy density.

## 4. Materials and Methods

Na_3_V(PO_4_)_2_/C was synthesized via a solid-state method. In a typical synthesis, the starting materials were 0.585 g NH_4_VO_3_ (5.00 mmol, Aladdin Reagent Co., Ltd., Shanghai, China), 1.321 g (NH_4_)_2_HPO_4_ (10.00 mmol, Sinopharm Chemical Reagent Co., Ltd., Shanghai, China), 0.874 g Na_2_CO_3_ (8.25 mmol, corresponding to 10% excess of Na, Sinopharm Chemical Reagent Co., Ltd., Shanghai, China), and 0.2 g acetylene black (AB, Sinopharm Chemical Reagent Co., Ltd., Shanghai, China), which served as the redundant and carbon source. All of the starting materials were mixed and then ball milled for 5 h at a speed of 400 rpm. The mixture was pressed into a pellet and then heated in a tube furnace in an Ar atmosphere at 350 °C for 6 h, after which the intermediate product was re-crushed and ball milled for 10 h and pressed into a pellet again. The final product was obtained by calcining the pellet in the tube furnace at 700 or 750 °C for 20 h. Occasionally, the final product was washed by 0.5 M HCl, 0.1 M NaOH, and deionized water successively in order to remove impurities.

XRD scans were carried out in a Rigaku Ultima IV powder X-ray diffractometer (Rigaku Corporation, Tokyo, Japan) using Cu K*α* radiation (λ = 1.5406 Å) operated at 40 kV and 30 mA from 2*θ* = 10–100° at a scan speed of 2°/min.

Time-of-fight (TOF) powder neutron diffraction data were collected using the VULCAN instrument from Spallation Neutron Sources (SNS), Oak Ridge National Laboratory (ORNL) [40]. Approximately 1.6 g of powder was filled into a vanadium sample can. An incident beam (5 mm × 12 mm) of 0.7 to 3.5 Å bandwidth, allowing 0.5~3.6 Å d-space in the diffracted pattern of the ±90° detector banks, was selected using the double-disk choppers at a 20 Hz frequency. High-resolution mode was employed with Δd/d ~0.25%. The SNS was at nominal, 1100 KW, power [40]. Powder neutron diffraction data were collected in high resolution mode for a duration of 3 h and processed using VDRIVE software [41]. Combined Rietveld refinement of XRD and ND data were performed using a GSAS code with the EXPGUI interface [42,43].

The chemical composition of the sample was determined using an Agilent ICP-MS/MS 8800 (Agilent Technologies, Santa Clara, CA, United States). The charge valence of vanadium was measured by vanadium K-edge XANES, which was collected in transmission mode at room temperature, using ion chamber detectors at beamline BL14W1 of the Shanghai Synchrotron Radiation Facility (SSRF) and a Si(111) double-crystal monochromator. The data were collected over an energy range from 200 eV below to 500 eV above the V (5465 eV). The incident photon energy was calibrated using standard V metal foil. Processing and fitting of the XANES data were performed using Athena software (version 0.9.25) [44].

The ^51^V ssNMR spectra were acquired on a Bruker Avance III 400 MHz NMR spectrometer (Bruker, Faellanden, Zurich, Switzerland) using 1.3 mm probehead at a spinning rate of 50 kHz. A recycle delay of 2 s and a 90° pulse length of 2 μs were used for spin echo. The chemical shift of ^51^V was referenced to V_2_O_5_ powder (−610 ppm).

The active materials, acetylene black (C_AB_), and polyvinylidene fluoride (PVDF), were mixed in the weight ratio of 8:1:1 using *N*-methyl-2-pyrrolidone (NMP) as the solvent. The obtained slurry was coated onto an Al foil substrate and dried overnight in a vacuum oven at 120 °C. The loading and thickness of the active material are ~3 mg/cm^2^ and ~20 μm, respectively. Cells were assembled in an argon-filled glove box using Na metal foil as the counter electrode (and reference electrode for three electrode cells), and a glass fiber as the separator. The electrolyte was composed of a solution of 1 M NaClO_4_ in propylene carbonate (PC) and fluoroethylene carbonate (FEC) (98:2 by volume). Cyclic voltammetry (CV) was performed using T-shaped Swagelok three electrode cells at a scan rate of 0.05 mV/s over the voltage range of 2.5–3.8 V and 4.3 V at 30 or 45 °C using a Versa STAT MV Multichannel potentiostat/galvanostat (Princeton Applied Research, Oak Ridge, TN, USA). Galvanostatic charge/discharge tests were performed using a coin cell at C/20 (i.e., 8.65 mA/g) in the voltage of 2.5–3.8 V and 2.5–4.3 V on a LAND CT-2001A (Wuhan, China) battery test system. QOCV was carried out by cycling the coin cell at C/20 for 30 min, followed by a 5 h relaxation between steps. For ex situ ^51^V ssNMR measurements, each cell was stopped at 3.8 and 4.3 V during the first charging and disassembled in an Ar-filled glove box. The electrodes were washed by PC and then dimethyl carbonate (DMC) for three times. The electrode materials were scraped carefully from the Al current collector and sealed in the probehead in the glove box.

## Supplementary Materials

The following are available online, Table S1: V- and Mn-based polyanionic cathodes with multielectron reactions, Table S2: Atomic parameters for Na_3_V(PO_4_)_2_, Figure S1: Crystal structure of Na_3_V(PO_4_)_2_, Figure S2: SEM image of Na_3_V(PO_4_)_2_, Figure S3: XANES spectra of Na_3_V(PO_4_)_2_ and Na_3_VCr(PO_4_)_3_, Figure S4: QOCV curve of the Na_3_V(PO_4_)_2_ cathode in the voltage range of 2.5–3.8 V, Figure S5: Cycling performance of the Na_3_V(PO_4_)_2_ cathode.
