# Supplementary material for: Recognition of V3+/V4+/V5+ Multielectron Reactions in Na3V(PO4)2: A Potential High Energy Density Cathode for Sodium-Ion Batteries
Source: Molecules. 2020 Feb 24;25(4):1000. doi: 10.3390/molecules25041000 (PMC7070626; doi:10.3390/molecules25041000)
Supplement: Supplementary file 1 [file molecules-25-01000-s001.pdf]

Supporting information

Recognition of $V^{3+}/V^{4+}/V^{5+}$ multielectron reactions in $Na_3V(PO_4)_2$: a potential high energy density cathode for sodium-ion batteries

Rui Liu,[1,2,] Ziteng Liang,[2] Yuxuan Xiang,[2] Weimin Zhao,[3] Haodong Liu,[4,*] Yan Chen,[5] Ke An,[5] Yong Yang[2,6,*]

1 School of Materials Science and Engineering, Shandong University of Science and Technology, Qingdao 266590, PR China.

2 Collaborative Innovation Center of Chemistry for Energy Materials, State Key Laboratory for Physical Chemistry of Solid Surface, Department of Chemistry, College of Chemistry and Chemical Engineering, Xiamen University, Xiamen, 361005, PR China.

3 College of Chemical Engineering and Safety, Binzhou University, Binzhou, 256603, PR China.

4 Department of NanoEngineering, University of California San Diego, 9500 Gilman Drive, La Jolla, California 92093, USA.

5 Neutron Scattering Division, Oak Ridge National Laboratory, Oak Ridge, TN, 37830, USA.

6 School of Energy Research, Xiamen University, Xiamen 361005, PR China.

* Yong Yang: yyang@xmu.edu.cn; Haodong Liu: haodong.liu.xmu@gmail.com

Table S1. V and Mn based polyanionic cathodes with multielectron reactions.

cathodes	redox couple	method	theoretical capacity (mAh/g)	theoretical energy density (Wh/kg)	Ref.
$Na_3MnTi(PO_4)_3$	$Mn^{2+}/Mn^{3+}/Mn^{4+}$	XPS	117	450	[1]
$Na_3MnZr(PO_4)_3$	$Mn^{2+}/Mn^{3+}/Mn^{4+}$	XPS	107	401	[2]
$Na_3VCr(PO_4)_3$	$V^{3+}/V^{4+}/V^{5+}$	XANES, ^{51}V NMR	117	439	[3]
$Na_3VAl(PO_4)_3$	$V^{3+}/V^{4+}/V^{5+}$	-	124	465	[4]
$Na_4VFe(PO_4)_3$	Fe^{2+}/Fe^{3+}, $V^{3+}/V^{4+}/V^{5+}$	Mössbauer spectra	166	548	[5]
$Na_4MnV(PO_4)_3$	$Mn^{2+}/Mn^{3+}/Mn^{4+}$, $V^{3+}/V^{4+}/V^{5+}$	-	167	601	[6-7]
$Na_3V_2(PO_4)_2F_3$	$V^{3+}/V^{4+}/V^{5+}$	sXAS, XANES	192	810	[8-9]
$Na_3V(PO_4)_2$	$V^{3+}/V^{4+}/V^{5+}$	^{51}V NMR	173	657	this work

Table S2. Atomic parameters for $Na_3V(PO_4)_2$.

Atom	Wyckoff site	*x*	*y*	*z*	Occupancy	100*U_{iso}
V	4*a*	0	0	0	1	2.33(7)
Na1	4*e*	0	0.0445(11)	0.25	1*	4.73(17)
Na2	8*f*	0.1704(4)	0.5375(7)	0.13424(17)	1*	2.52(10)
P	8*f*	0.16835(28)	0.5218(5)	0.38712(12)	1	2.12(6)
O1	8*f*	0.1658(5)	0.3890(8)	0.28908(25)	1	3.44(12)
O2	8*f*	0.1114(4)	0.3264(8)	0.45872(23)	1	2.50(11)
O3	8*f*	0.0811(4)	0.7863(7)	0.38498(26)	1	2.46(10)
O4	8*f*	0.3306(4)	0.5925(7)	0.41351(26)	1	2.13(10)

* The occupancy of Na is slightly larger than 1, which may be caused by calculating error. Consequently, the value is fixed to 1 during the refinement.

Table S3. Selected bond distance (Å) for $Na_3V(PO_4)_2$.

V-O2	2.020(4)	Na2-O1	2.274(5)
V-O3	2.072(4)	Na2-O1’	2.532(6)
V-O4	1.986(4)	Na2-O2	2.573(5)
P-O1	1.514(5)	Na2-O2’	2.797(5)
P-O2	1.498(5)	Na2-O3	2.608(6)
P-O3	1.550(5)	Na2-O3’	2.616(6)
P-O4	1.553(5)	Na2-O4	2.336(5)
Na1-O1	2.353(6)	Na2-O4’	2.872(5)
Na1-O3	2.382(5)		
Na1-O4	2.778(4)		

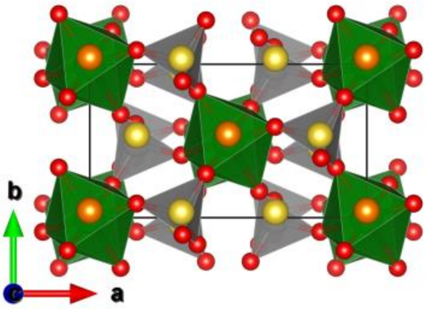

Figure S1. Crystal structure of $Na_3V(PO_4)_2$.

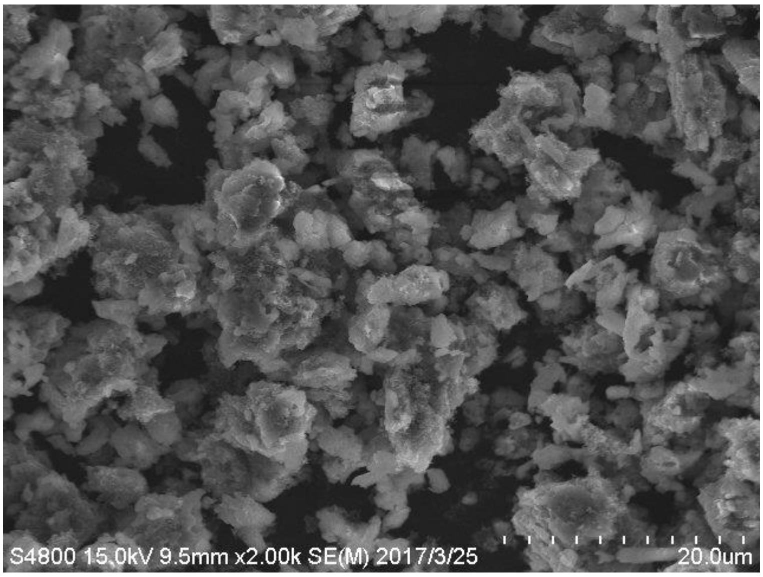

Figure S2. SEM image of $Na_3V(PO_4)_2$.

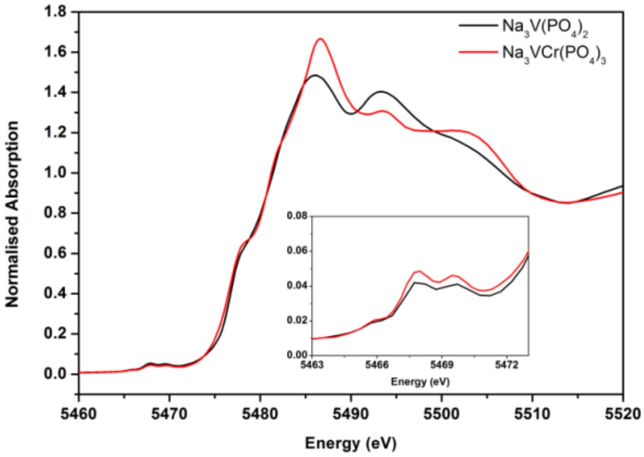

Figure S3. XANES spectra of of $Na_3V(PO_4)_2$ and $Na_3VCr(PO_4)_3$.

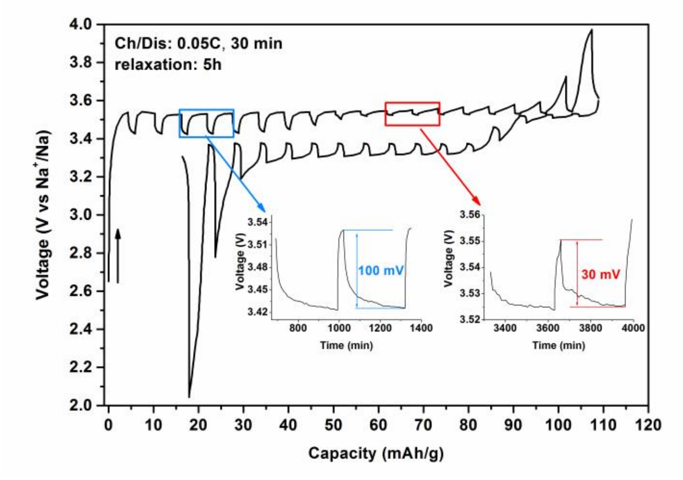

Figure S4. QOCV curve of $Na_3V(PO_4)_2$ cathode in the voltage range of 2.5 – 3.8 V.

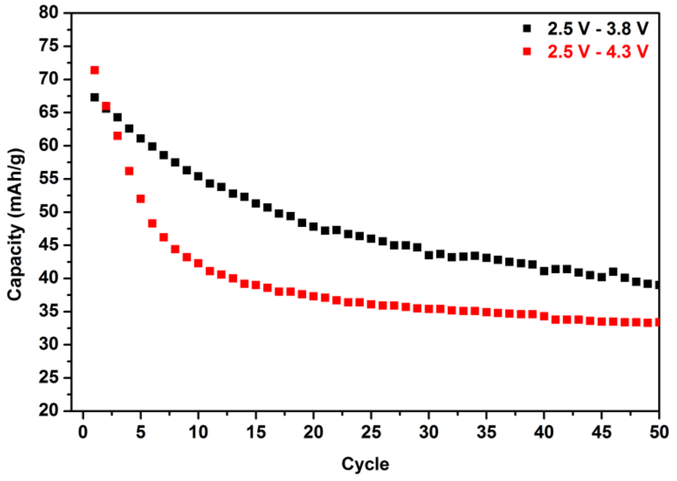

Figure S5. Cycling performance of $Na_3V(PO_4)_2$ cathode.

References

(1) Gao, H. C.; Li, Y. T.; Park, K.; Goodenough, J. B., Sodium Extraction from NASICON-Structured $Na_3MnTi(PO_4)_3$ through Mn(III)/Mn(II) and Mn(IV)/Mn(III) Redox Couples. *Chem Mater* **2016,** *28*, 6553-6559.

(2) Gao, H. C.; Seymour, I. D.; Xin, S.; Xue, L. G.; Henkelman, G.; Goodenough, J. B., $Na_3MnZr(PO_4)_3$: A High-Voltage Cathode for Sodium Batteries. *J Am Chem Soc* **2018,** *140*, 18192-18199.

(3) Liu, R.; Xu, G. L.; Li, Q.; Zheng, S. Y.; Zheng, G. R.; Gong, Z. L.; Li, Y. X.; Kruskop, E.; Fu, R. Q.; Chen, Z. H.; Amine, K.; Yang, Y., Exploring Highly Reversible 1.5-Electron Reactions ($V^{3+}/V^{4+}/V^{5+}$) in $Na_3VCr(PO_4)_3$ Cathode for Sodium-Ion Batteries. *Acs Appl Mater Interfaces* **2017,** *9*, 43632-43639.

(4) Lalere, F.; Seznec, V.; Courty, M.; David, R.; Chotard, J. N.; Masquelier, C., Improving the energy

density of $Na_3V_2(PO_4)_3$-based positive electrodes through V/Al substitution. *J Mater Chem A* **2015,** *3*, 16198-16205.
(5) de Boisse, B. M.; Ming, J.; Nishimura, S. I.; Yamada, A., Alkaline Excess Strategy to NASICON-Type Compounds towards Higher-Capacity Battery Electrodes. *J Electrochem Soc* **2016,** *163*, A1469-A1473.
(6) Zakharkin, M. V.; Drozhzhin, O. A.; Tereshchenko, I. V.; Chernyshov, D.; Abakumov, A. M.; Antipov, E. V.; Stevenson, K. J., Enhancing Na^+ Extraction Limit through High Voltage Activation of the NASICON-Type $Na_4MnV(PO_4)_3$ Cathode. *ACS Applied Energy Materials* **2018,** *1*, 5842-5846.
(7) Chen, F.; Kovrugin, V. M.; David, R.; Mentré, O.; Fauth, F.; Chotard, J. N.; Masquelier, C., A NASICON-Type Positive Electrode for Na Batteries with High Energy Density: $Na_4MnV(PO_4)_3$. *Small Methods* **2018,** *2*, 1800218.
(8) Yan, G. C.; Mariyappan, S.; Rousse, G.; Jacquet, Q.; Deschamps, M.; David, R.; Mirvaux, B.; Freeland, J. W.; Tarascon, J. M., Higher energy and safer sodium ion batteries via an electrochemically made disordered $Na_3V_2(PO_4)_2F_3$ material. *Nat. commun.* **2019,** *10:585*, 1-12.
(9) Nguyen, L. H. B.; Broux, T.; Camacho, P. S.; Denux, D.; Bourgeois, L.; Belin, S.; Iadecola, A.; Fauth, F.; Carlier, D.; Olchowka, J.; Masquelier, C.; Croguennec, L., Stability in water and electrochemical properties of the $Na_3V_2(PO_4)_2F_3$ – $Na_3(VO)_2(PO_4)_2F$ solid solution. *Energy Storage Mater* **2019,** *20*, 324-334.
